# *Anvillea garcinii* extract inhibits the oxidative burst of primary human neutrophils

**DOI:** 10.1186/s12906-016-1411-7

**Published:** 2016-11-03

**Authors:** Hanane Boukemara, Margarita Hurtado-Nedelec, Viviana Marzaioli, Dalila Bendjeddou, Jamel El Benna, Jean-Claude Marie

**Affiliations:** 1 INSERM, U1149, CNRS-ERL8252, Centre de Recherche sur l’Inflammation (CRI), 16 rue Henri Huchard, 75018 Paris, France; 2 Université Paris Diderot, Sorbonne, 75018 Paris, France; 3 Laboratoire d’Excellence Inflamex, Faculté de Médecine, Site Xavier Bichat, 75018 Paris, France; 4 AP-HP, HUPNVS Bichat-Claude Bernard, UF Dysfonctionnements Immunitaires, 75018 Paris, France; 5 Laboratoire de Biologie, eau et Environnement - Département de Biologie, Faculté SNV/ STU, Université 8 mai 1945 - Guelma, BP 401, Guelma, 24000 Algeria

**Keywords:** Inflammation, NADPH oxidase, *Anvillea garcinii*, *Zygophyllum gaetulum*, North Africa

## Abstract

**Background:**

*Anvillea garcinii* Coss. & Durieu (*Anv*) plant is used as a traditional North African medicine against several diseases associated with inflammation. At inflammatory sites, reactive oxygen species (ROS) produced in excess by activated phagocyte NADPH oxidase (NOX2) can accentuate inflammatory responses. Thus, we investigated if *Anv*-water soluble polysaccharides could modulate primary human neutrophil oxidative burst in vitro.

**Methods:**

Human neutrophils were isolated from fresh whole blood and O_2_^.-^ generation was measured by cytochrome *c* reduction assays. Western blots were used to analyse the translocation of PKC, p47^phox^ (a key component of NOX2 activity) to neutrophil plasma membrane. Also, myeloperoxidase (MPO) release in the extracellular medium was studied by western blots. Flow cytometric analysis was used to detect CD11b membrane expression.

**Results:**

Water soluble polysaccharides from *Anv* dose-dependently inhibited N-formyl-methionyl-leucyl-phenylalanine (fMLF)- and phorbol myristate acetate (PMA)-induced O_2_^.-^ generation by human neutrophils. Moreover, *Anv*-polysaccharides strongly inhibited PMA-induced PKCβ and p47^phox^ translocation to membranes and p47^phox^ phosphorylation on Ser328, a main PKC target. In contrast, polysaccharides extract from *Zygophyllum gaetulum* plant, which is also used as a traditional North African medicine against inflammatory diseases, was ineffective on this PKCβ-p47phox pathway. Further, *Anv* inhibited important neutrophil degranulation markers corresponding to myeloperoxidase (MPO) release and CD11b membrane expression.

**Conclusion:**

The process of down-regulating NADPH oxidase by polysaccharides extracts from *Anv* provides new insights into the mechanism of *Anv*’s anti-inflammatory actions.

**Electronic supplementary material:**

The online version of this article (doi:10.1186/s12906-016-1411-7) contains supplementary material, which is available to authorized users.

## Background

Increased reactive oxygen species (ROS) can damage healthy bystander tissues, thereby contributing to several inflammatory diseases such as rheumatoid arthritis and inflammatory bowel diseases [1]. Neutrophil is the main ROS producing phagocyte which play an essential role in host defence against microbial pathogens and in inflammation. In response to stimulating agents such as the bacterial peptide *N*-formyl-methionyl-leucylphenyl-alanine (fMLF), neutrophils release large amounts of superoxide anion (O_2_^.-^) and other reactive oxygen species (ROS) such as hydrogen peroxide (H_2_O_2_), used by myeloperoxidase (MPO) to produce hypochloric acid, in a phenomenon called the respiratory burst [2]. Nicotinamide adenine dinucleotide phosphate (NADPH) oxidase or NOX2, the enzyme responsible for superoxide anion production by phagocytes, is a multicomponent enzyme system consisting of membrane associated cytochrome b558 (composed of gp91^phox^ and p22^phox^) and the cytosolic components p47^phox^, p67^phox^, p40^phox^, and rac1/2 (a small GTPase) [3, 4]. Neutrophil activation leads to phosphorylation of the cytosolic components such as p47^phox^, a protein which plays an important role in the assembly and activation of NADPH oxidase.

Activated neutrophils and several associated products such as ROS, myeloperoxidase (MPO), elastase and inflammatory adipokines have been found in synovial fluid of patients with rheumatoid polyarthritis [5, 6]. Neutrophils are highly mobile cells which are densely packed with secretory granules. Degranulation, a mechanism independent of ROS production, can also be incriminated as being a main cause of pulmonary disorders such as severe asphyxic episodes of asthma [7]. The dual role of neutrophils has been further highlighted during respiratory viral infection whereby it contributed to beneficial antiviral responses as well as detrimental tissue pathology and cellular inflammation [8]. Thus, the inhibition of excessive oxidant production and degranulation represent an important therapeutic target and could in part explain the beneficial effects of certain natural products used against inflammatory disorders. To this end, bioactive products isolated from medicinal plant such as *Garcinia buchananii* and *Ficus aurantiaca* Griff have been shown to be antioxidative by respectively displaying in vitro H_2_O_2_ scavenging activity [9] or inhibiting neutrophil ROS production [10]. Indeed, integration of the wealthy African medication for several diseases including inflammation in western medicine is considered as a promising challenge [11]. Medicinal plant extracts are usually made with water and water-soluble polysaccharides isolated from plants have attracted much attention because of their broad spectrum of therapeutic properties and relative low toxicity [12].

In this context, we investigated if water-soluble polysaccharide-extract from an endemic plant which grows in northern Africa and particularly in Morocco and Algeria, *Anv*, could have anti-inflammatory effects. This plant is known by traditional healers to possess several medicinal properties in North-African countries [13]. For example, their crude extracts as well as their infusions are widely used to treat colds, diabetes, stomach and liver diseases often associated with inflammation and they are also antiseptic and antispasmodic [14–16].

Our study shows that *Anvillea garcinii* Coss. & Durieu water-soluble polysaccharide extract was efficient in inhibiting ROS production and degranulation of primary human phagocytes. Indeed, *Anv* was efficient in inhibiting neutrophil superoxide anion induced by PMA or fMLF. Analysis of the mechanism indicated a selective inhibition of a strong PKC phosphorylation site, Ser 328 on p47^phox^ crucial for the assembly and function of neutrophil NOX2.

## Methods

### Reagents

Phorbol myristate acetate (PMA), formyl-methionyl-leucylphenylalanine (fMLF), Tween 20, luminol, cytochrome *c*, horse radish peroxidase (HRP), phosphatases inhibitors and proteases inhibitors, ATP, HBSS, acetylsalicylic acid and PBS and other chemicals were purchased from Sigma Aldrich (Saint-Quentin Fallavier, France). Dextran T500 and Ficoll were purchased from GE Healthcare (Orsay, France). HEPES was from life technologies (Saint Aubin, France). SDS-PAGE (sodium dodecyl sulphate-polyacrylamide gel electrophoresis), and Western blot were purchased from Bio-Rad (Richmond, CA, USA). Antibodies against protein kinase C were from GeneTex (Irvine, CA, USA). Antibodies against, p22, βactin as well as secondary HRP-labeled goat anti-rabbit antibodies were from Santa Cruz Biotechnology (Heidelberg, Germany). The anti-p47^phox^ antibody was kindly provided by Dr. B. M. Babior (The Scripps Research Institute, La Jolla, CA, USA). The use of our rabbit polyclonal antibodies against phospho-sites p47^phox^ (phospho-Ser328) have been described previously [17].

### *Anvillea garcinii* collection and preparation of water-soluble polysaccharide extract

*Anvillea garcinii* Coss. & Durieu (*Anv*) was collected from Biskra Ghardaya (north of Algerian Sahara) and was taxonomically authenticated by Dr. Ali Zitouni (University 8 Mai 1945 of Guelma, Algeria) and deposited at the herbarium of the university with the voucher specimen; (No.A.g.2010-1). A standardized water-soluble polysaccharide extract was prepared as described previously [18]. Briefly, 170 g of *Anv* powder, corresponding to the plant aerial part, was suspended in distilled water (1 g/20 mL) and stirred for 3 h at 100 °C before cooling overnight at 8 °C. The supernatant obtained by centrifugation for 30 min at 4000 g was reduced to half volume by evaporation. Polysaccharides were precipitated from the filtrates by the adding four volumes of ethanol (95 %). The precipitates (16.48 g = 9.7 %) were collected by centrifugation for 30 min at 15,000 g and washed with ethanol and dried. The sample was dissolved in distilled water (1 g/50 mL), dialysed against distilled water for 72 h at 4 °C. The dialysis residue was treated with tri-chloro-acetic acid and sodium acetate to remove proteins. The crude polysaccharide mixtures were precipitated by ethanol and air dried. The percentage yield relative to dry samples weight was 3.18 % and the presence of proteins was not detected by colorimetric methods [19, 20]. Similarly, we also extracted water-soluble polysaccharides (2.48 % yield) from another identified traditional medicinal plant from the same region, *Zygophyllum gaetulum* Emb. & Maire (*Zyg*), [21]. It was collected and identified by by Dr. Ali Zitouni and deposited at the above herbarium with the voucher (No.A.g.2010-2).

### Neutrophil isolation from peripheral human blood

Freshly drawn blood from healthy subjects was used to isolate neutrophils by dextran sedimentation and Ficoll fractionation [22]. After centrifugation at 400 g for 30 min, neutrophil and monocyte/lymphocyte rings were collected. Erythrocytes were removed from neutrophils by hypotonic lysis. Following isolation, neutrophils were suspended in appropriate medium, such as Hank’s balanced salt solution (HBSS). Cell purity and counting were determined by flow cytometry analysis (FACS Canto II cytometer (BD, San Jose, CA, USA) and Turks’ blue staining respectively. The viability of neutrophils with or without the polysaccharides was also determined by differential permeability of DNA-binding dyes using the ViaCount reagent by ^Guava^ easy Cyte (IFAS-Cytometry Bioscience, Millipore, St Quentin en Yvelines, France).

### Measurement of superoxide production

To measure superoxide anion or O_2_^.-^ production, isolated neutrophils cells (5 × 10^5^) were suspended in 500 μL of HBSS containing 1 mg/mL cytochrome *c* in the presence or absence of different concentrations of *Anv*. The samples were placed in the thermostated chamber of a spectrophotometer (Uvikon, Thermo Fisher, Villebon, France) preheated at 37 °C. After 15 min, cells were stimulated with 10^−6^ M of N-formyl-methionyl-leucyl-phenylalanine (fMLF) or 100 ng/mL of phorbol myristate acetate (PMA). Changes in absorbance were measured at 550 nm for 10 min.

### Evaluating *Anv* scavenging activity of O_2_^.-^

To evaluate O_2_^.-^ scavenging activity, the O_2_^.-^ generation substrate xanthine (200 μM) and cytochrome *c* (1 mg/mL) were incubated with different concentrations of polysaccharides of *Anv* at 30 °C during 15 min. The enzyme xanthine oxidase was added and the amount O_2_^.-^ was measured by assaying the cytochrome *c* reduction at 550 nm in a spectrophotometer (Uvikon) for 10 min.

### Effect of *Anv* on neutrophil degranulation

Neutrophils (5 × 10^6^ per 500 μL HBSS) were incubated in the presence or the absence of different concentrations of *Anv* for 15 min at 37 °C. Then, they were stimulated with either 10^−6^ M fMLF for 5 min or 100 ng/mL PMA for 15 min. Degranulation was stopped by cooling cell aliquots in ice-cold methanol (−80 °C) for 5 s. Cells were then centrifuged (4 °C) for 8 min at 400 *g*. Supernatants were removed and denatured with Laemmli sample buffer [23] for western blot analysis.

### Flow cytometric analysis of the CD11b expression at neutrophil surface

Isolated neutrophils (2.5 × 10^6^ per 500 μL PBS) were incubated at 37 °C for 15 min in the presence or the absence of *Anv* extracts. Samples were then stimulated with fMLF (10^−6^ M) for 5 min. The reaction was stopped by cooling the cell aliquots on ice. 100 μL of each sample was then stained with 20 μL of PE-conjugated anti-human CD11b monoclonal antibody (BD Biosciences, San Jose, CA) for 15 min at room temperature in the dark. Then the cells were washed twice with PBS at room temperature and analyzed by flow cytometry using FACS Canto II cytometer (BD Biosciences, San Jose, CA, USA) as described [6].

### PKCβ and p47^phox^ translocation to neutrophils plasma membrane

The experiment was performed as described before [17], briefly neutrophils (50 × 10^6^ per mL) in HBSS buffer were incubated for 15 min at 37 °C in the absence (control) or the presence of either *Anv* or *Zyg* extract at 300 ug/mL and then cells were stimulated with 100 ng/mL PMA for 8 min. The reaction was stopped by adding 4 volumes of ice-cold PBS and cells were pelleted by centrifugation at 400 *g* for 8 min at 4 °C. The suspended cells were disrupted by sonication and centrifuged at 400 *g* for 8 min. The postnuclear supernatant was loaded onto a discontinuous sucrose gradient (35 % sucrose, 15 % sucrose), centrifuged for 45 min at 150,000 *g*. The membrane fraction in the 15 % sucrose layer was collected and washed in PBS before denaturing in Laemmli sample buffer. The samples were boiled for 3 min and stored at -80 °C until use.

### Western blot analysis

The different samples were subjected to 10 % SDS-PAGE using standard techniques [23]. The separated proteins were transferred to nitrocellulose, which was blocked with 5 % non-fat dry milk in Tris-buffered saline with 0.1 % tween-20 (TBST) for 1 h. After blocking, the nitrocellulose membranes were incubated for 1 h at 37 °C or overnight at -4 °C with specific antibodies (Anti MPO (1:5000), anti-phosphoSer328-p47^phox^ (1:2000), anti-p47^phox^ (1:5000), anti-PKCβ or α (1:1000)) followed by three washes with TBST, the membranes were incubated with HRP-labelled goat anti-rabbit or anti-mousse antibody (1:5000). After three washes with TBST, revelation was performed by a chemiluminescence method (luminol reagent, Santa Cruz Biotechnology, Santa Cruz, CA), according to the manufacturer instructions and using Amersham Imager 600 (LifeSciences, Velizy, France). The intensity of bands was quantified by densitometry using the Image J analysis program (National Institute of Health, USA). Alternatively, the membranes were incubated with an alkaline phosphatase-conjugated goat anti-mouse or goat anti-rabbit and proteins were revealed with the NBT/BCIP reagents (Saint Louis, Missouri, USA) in the carbonate buffer (0.1 mM NaHCO_3_, 1 mM MgCl_2_, pH 9.8).

### Statistical analysis

All results are expressed as mean ± SEM. One-way analysis of variance with the Tukey-Kramer post hoc test for multiple comparisons was implemented using GraphPad Prism version 6.0 for Windows (GraphPad Software, San Diego, CA).

## Results

### *Anv* inhibited superoxide production by neutrophil in response to PMA

**Fig. 1:**
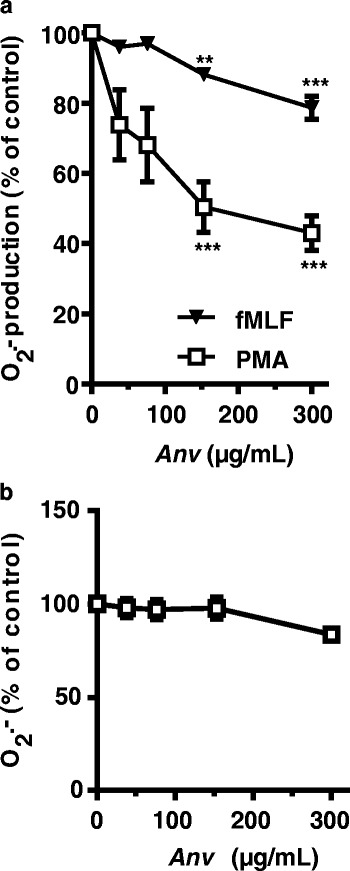
Effect of *Anv* on superoxide anion production by human neutrophils. Human neutrophils were incubated at 37 °C during 15 min in the presence or the absence of different concentrations of *Anv* (**a**). Then they were stimulated with either fMLF (10^−6^ M) or PMA (100 ng/mL) and simultaneously, superoxide production was measured using the cytochrome *c* reduction assay at 550 nm in a spectrophotometer for 10 min. The amount of superoxide produced is expressed as a percentage of values obtained when neutrophils were stimulated with fMLF or PMA alone. In **b**, we investigated if *Anv* extract can scavenge superoxide anion. The O_2_^.-^ generation substrate xanthine and cytochrome *c* were incubated with different concentrations of *Anv* extract at 30 °C during 15 min. The enzyme xanthine oxidase was added and the amount O_2_^.-^ was measured by cytochrome *c* reduction assay. All results are means ± SEM of three or more separate experiments. ** *P* < 0.01, *** *P* < 0.0001

The effect of *Anv* on ROS production by human neutrophils was compared with that of *Zyg*, another North African medicinal plant *Zyg*, which is similarly used to treat against inflammatory diseases, even though they are of different genus. We also studied the established anti-inflammatory drug, aspirin [10] and fMLF or PMA was used to stimulate ROS production which was measured by using luminol-amplified chemiluminescence as shown in Additional file 1. *Anv* was a strong inhibitor of ROS production with a similar IC_50_ value (60 μg/mL) for either fMLF or PMA response while *Zyg* was poorly effective. The aspirin IC_50_ values were 200 μg/mL and 300 μg/mL for inhibiting fMLF or PMA response respectively. To gain further insight in the mechanism of *Anv* inhibition, we explore its effect on the production of superoxide anion, the first product of NADPH oxidase. We used the cytochrome *c* reduction assay which is a specific technique for measuring superoxide anion. As shown in (Fig. 1a) *Anv* dose-dependently inhibited O_2_^.-^ production by human neutrophils stimulated with fMLF. Further, a dramatic inhibition of O_2_^.-^ production was observed in response to PMA. We investigated if this inhibition was due to a scavenging of O_2_^.-^ by *Anv* extract by using the in vitro xanthine oxidase production of O_2_^.-^ . Results in (Fig. 1b), show that the production of O_2_^.-^ was not significantly altered in the presence of increasing concentrations of *Anv*. Taken together, the results are in favour of an *Anv* effect on NADPH oxidase activity as opposed to a scavenging activity.

### *Anv* inhibited p47^phox^ and PKC translocation to the neutrophil plasma membrane in response to PMA

**Fig. 2:**
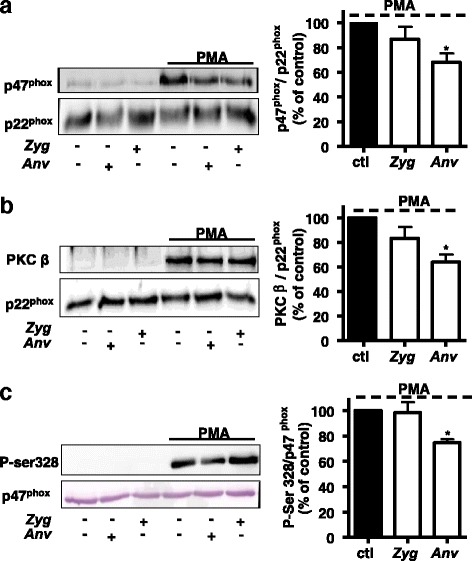
Effect of *Anv* and *Zyg* on PKC and p47^phox^ translocation and on p47^phox^ phosphorylation in human neutrophils. In figures **a**, **b** and **c**, neutrophils were incubated for 15 mins at 37 °C either without or with 300 μg/ml of water-soluble extracts from *Anv* or Zygophyllum gaettulum (*Zyg*), followed by PMA stimulation for 8 min. *Zyg* has no significant effect on phagocyte O_2_^.-^ generation and represents a control versus *Anv*. Cells were lysed and membranes were purified as described in Methods and analyzed by SDS-PAGE and Western blot with anti-p47^phox^ (**a**) or anti-PKCβ (**b**) and anti-p22^phox^ Ab (p22^phox^) antibodies. Western blots from different experiments were scanned and the intensity of p47^phox^ and PKCβ translocation was expressed relative to the protein amount of p22^phox^. The cumulated data is shown in the histogram as a percentage to control (PMA alone). In figure **c**, immunoblotting was performed with anti-phospho-Ser328 Ab, or anti-p47^phox^ Ab (p47^phox^). The cumulated data is shown in the histogram as a percentage to control (PMA alone). All the results are expressed as means ± SEM of three or more separate experiments. * *P* < 0.05, compared to control values

We investigated if *Anv* can modulate PMA-induced translocation of p47^phox^ and PKC in neutrophils which represents a potential immuno-modulatory pathway. We compared the effect of *Anv* with the water-soluble polysaccharide extract of *Zyg* which did not modulate O_2_^.-^ production by human neutrophils in response to PMA, thus suggesting the involvement of different mechanisms and represented a potential control versus active *Anv* (data not shown). The results in (Fig. 2a, left panel) show that neither *Anv* nor *Zyg* was able to promote the translocation of p47^phox^ to neutrophil plasma membrane in the resting state. When neutrophils were incubated with PMA, it stimulated a strong translocation of p47^phox^. This PMA-induced translocation was significantly inhibited in the presence of *Anv* and not *Zyg* as indicated by the histogram. Similar results were obtained with PKC*β* showing that *Anv* was effective in inhibiting PKC*β* translocation to plasma membrane in response to PMA (Fig. 2b). Further, activation of NADPH oxidase in neutrophils by PMA is known to be accompanied by a strong phosphorylation of Ser328 in the regulatory subunit, p47^phox^ [24]. We detected little or no phosphorylation using a specific anti-phosphoSer328 antibody, in control samples (Fig. 2c), while PMA induced a strong Ser328 phosphorylation. Interestingly, *Anv* at the same concentration used above (300 μg/ml) significantly inhibited this phosphorylation of p47^phox^ on Ser328 stimulated by PMA while *Zyg* was ineffective.

### *Anv* inhibited neutrophil degranulation

**Fig. 3:**
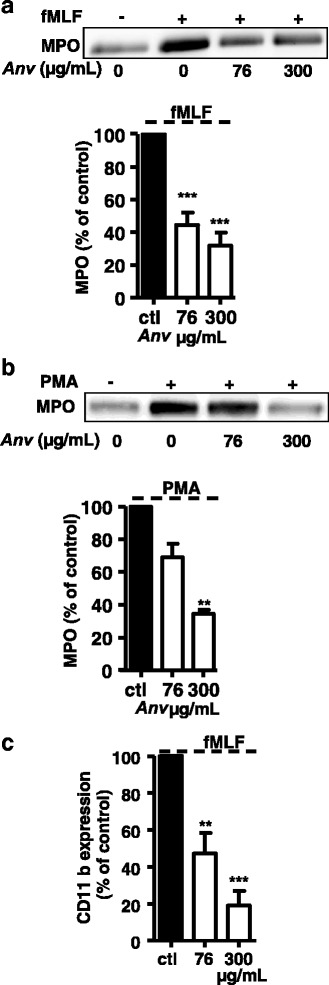
Effect of *Anv* on fMLF- or PMA-induced degranulation by human neutrophils. Human neutrophils were incubated in the absence or the presence of different concentrations of *Anv* extract and then stimulated with either fMLF (**a**) for 5 min or PMA (**b**) for 15 min. Cells were pelleted (4 °C) at 8000 g for 30 s and the extracellμar medium was centrifuged at 10,000 g during 12 min before analysis by SDS-PAGE and Western blot using anti-MPO antibody. The western blots from different experiments were scanned and the intensity of MPO was quantified by densitometry analysis. The histogram shows the percentage of MPO density as compared to control values with either fMLF or PMA only. In figure **c**, human neutrophils were similarly incubated as above either without or with *Anv* extract and then stimμated with fMLF for 5 min. CD11b expression at neutrophil surface was evaluated using a PE-conjugated anti-human CD11b monoclonal antibody and flow cytometric analysis as described in Methods. Histogram shows the percentage of CD11b expression relative to fMLF response. All results are expressed as means ± SEM of three or more separate experiments. ** *P* < 0.01, *** *P* < 0.0001, compared to control

We investigated whether *Anv* extract could affect other important neutrophil functions such as degranulation. To this end, neutrophils were incubated without (control) and with 76 to 300 μg/mL of *Anv* extracts and then stimulated with fMLF or PMA as described in methods. Then myeloperoxidase (MPO), the most abundant pro-inflammatory enzyme stored in azurophilic granules of neutrophils, was detected in the extracellular medium by using western blot analysis. The results (Fig. 3a and b) show that *Anv* was effective in inhibiting MPO release in the extracellular medium induced by either fMLF or PMA. Further, we analysed the effect of *Anv* on fMLF-induced degranulation of specific granules by measuring the neutrophil surface expression of CD11b. In (Fig. 3c), the data of flow cytometric analysis are presented as a percentage of control values (fMLF only) and showed that *Anv* inhibited CD11b expression at the neutrophil outer membrane. Taken together, the results suggest that *Anv* inhibits neutrophil degranulation processes.

## Discussion

Innate immunity serves as an essential first-line of defense against microbial pathogens and foreign substances. Phagocytic cells such as neutrophils play a key role in innate immunity because of their ability to recognize, ingest and destroy pathogens by oxidative and nonoxidative mechanisms. However, neutrophil hyper-activation has been shown to induce tissue injury and inflammatory reactions [1, 8]. Thus, inhibition of neutrophil hyper activation represents an interesting strategy to develop novel anti-inflammatory agents. There is increasing research in identifying novel natural plant products to integrate in conventional immunomodulators as they often have less side effects [11, 12]. Our data showed that water-soluble polysaccharides extract isolated from medicinal plants, *Anv* inhibited fMLF- and PMA-induced superoxide anion (O_2_^.-^) production in human neutrophils. *Anv* inhibited PMA-induced PKC activation resulting in the inhibition of PMA-induced phosphorylation and translocation of the NADPH oxidase subunit p47^phox^. Also, the *Anv* extract inhibited MPO degranulation. These results suggest that polysaccharides isolated from *Anv* could exert a strong anti-inflammatory effect by inhibiting neutrophil functions and by limiting reactive oxygen species (ROS) propagation to nearby tissues.

An important difference in the inhibitory effect of *Anv* was observed on O_2_^.-^ produced in response to either fMLF and PMA. The pronounced effect on PMA versus fMLF may be due to different mechanisms including O_2_^.-^ scavenging since ROS-scavenging activity has been observed with some polysaccharide extracts [25, 26]. Polysaccharide isolated from *Artemisa tripartite* has been shown to scavenge both H_2_O_2_ and O_2_^.-^ and their capacity to scavenge may be due to difference in polysaccharide composition and size [27]. The potent scavenging property of exogenous polysaccharides compounds represents a therapeutic potential for oxidative stress-associated inflammatory diseases. This has been recently highlighted in vivo by endogenous scavenging lectin such as Reg3 alpha which suppresses extracellular reactive oxygen species to protect from liver toxicity [28]. In our study, we found that O_2_^.-^ was not scavenged by *Anv* polysaccharide using an in vitro assay whereby superoxide anion (O_2_^.-^) is produced by xanthine oxidase [27].

We thus explored how *Anv* could modulate neutrophil extracellular production of O_2_^.-^ by NADPH oxidase (NOX2) since several polysaccharide extracts have been shown to modulate phagocyte ROS production without or with scavenging activity [29, 30]. Interestingly, it was shown that while water-insoluble chitosan derivatives can activate neutrophils by a phagocytosis mechanism, soluble ones would prime neutrophils and potentiate oxidative burst [29]. Thus, soluble polysaccharide extracts can have both stimulatory and inhibitory effects on neutrophil functions. In our study, it is unlikely that water-soluble polysaccharide extract of *Anv* directly inhibited the NOX2 enzyme since there was a preferential inhibition of O_2_^.-^ production by NOX2 induced by two different stimulators, PMA or fMLF. Moreover, PMA represents not only a positive control but it is an appropriate tool to explore the signal transduction pathways involved in the activation of NOX2 and degranulation process of neutrophils [24, 31, 32]. PMA can directly activate the phospholipid-dependent protein kinase C, which translocate to the plasma membrane and by phospohorylating NADPH oxidase components such as p47^phox^ participates in the assembly and activation of NOX2 [33]. All PKC isoforms present in human neutrophils, including PKCβ are known to activate NADPH oxidase [24]. The biological relevance of PKC pathway was sustained by the use of opsonised zymosan, components of yeast cell wall, which activated a PKC-dependent activation of NOX2 in neutrophils [34]. We found that *Anv* inhibited the membrane translocation of p47^phox^ and PKCβ induced by PMA. Water-soluble polysaccharide extract of another North African medicinal plant *Zyg*, which is similarly used as *Anv* against inflammatory diseases, was also studied in order to explore their mechanisms. Further, *Zyg* is sometimes given together with *Anv* as traditional medicine. In contrast to *Anv*, we found that *Zyg* barely inhibited O_2_^.-^ production by neutrophils in response to either PMA or fMLF (data not shown). In line with this observation, we found that *Zyg* was ineffective in modulating the membrane translocation of p47^phox^ and PKCβ induced by PMA. The production of superoxide anions by NOX2 in human neutrophil is accompanied by extensive phosphorylation of p47^phox^ which is an excellent substrate for PKC [24]. To further validate the action of *Anv* on PKC/ p47^phox^, we studied its effect on serine 328 of p47^phox^, which is one of the most phosphorylated serine residues by PKC. The results obtained by using our raised antibody against this phosphorylated residue showed that *Anv* polysaccharide was able to inhibit PMA-induced phosphorylation of serine 328 of p47^phox^ as well as its translocation to neutrophil plasma membrane and *Zyg* extract was ineffective. Altogether, the results indicate a clear effect of water-soluble *Anv* polysaccharide extract on the modulation of PMA-induced superoxide anions.

The progression of inflammation is significantly affected by myeloperoxidase (MPO) released from azurophilic granules of neutrophils accumulated at inflammatory sites [35]. MPO-dependent oxidative system can damage host tissue through the generation of oxidants. There are some data suggesting that polysaccharides may influence the traffic of different granules in neutrophils and their degranulation in phagosomes or the extracellular environment. It has been recently shown that water soluble polysaccharides extracted from *Bupleurum chinense* inhibited fMLF-induced HL60 cell recruitment and involved the inhibition of rac-1 activation [36]. The traffic of cytosolic rac-1 or rac-2 to plasma membrane is crucial in the assembly of functional NADPH oxidase and may represent a site of inhibitory action for polysaccharide extracts. Moreover, rac-2 serves has a selective role in the degranulation of neutrophils, a process which is independent of ROS production [37]. Further, the anti-inflammatory aqueous extract of *Punia granatum* has been shown to selectively inhibit MPO activity of human neutrophils which depends on degranulation process [38]. In contrast, it has been shown that acidic polysaccharides isolated from the medicinal plant *Tanacetum vulgare* L. can enhance neutrophil MPO release [39]. Thus, it is important to test the effect of *Anv* on neutrophil degranulation initiated by fMLF or PMA. The effect on azurophilic or specific granules was estimated by following MPO and CD11b expression respectively. We could show that *Anv* extract was more effective in inhibiting fMLF- than PMA-induced MPO release in the extracellular medium. Also, the surface expression of CD11b, a sensitive marker of PMN activation by fMLF was inhibited by *Anv*. Our results indicate that *Anv* polysaccharide can inhibit both PMA and fMLF induced degranulation processes which involve a large group of intracellular signalling molecules [40].

## Conclusion

**Fig. 4:**
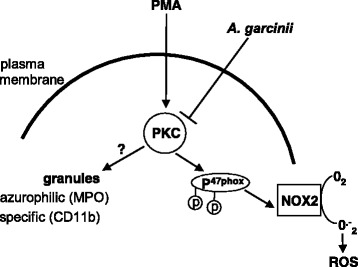
Mechanism of action of *A. garcininii* (*Anv*)*.* Polysaccharides extract from *Anv* strongly inhibits neutrophils response induced by phorbol myristate acetate (PMA) an activator of Protein Kinase C (PKC). Activation of PKC leads to specific phosporylation of p47^phox^ and its translocation to plasma membrane to activate NADPH oxidase (NOX2) which produces ROS. *Anv* prevents this activation by inhibiting PKC and thus preventing p47^phox^ phosphorylation and its translocation. Further, *Anv* by its inhibitory action on PKC, is also able to counteract the degranulation of azurophilic and specific granules of activated neutrophils

We demonstrated that *Anv* polysaccharides have a strong antioxidant action on neutrophils as summarized in Fig. 4. Moreover, *Anv* was very efficient at limiting NADPH oxidase activation by inhibiting the phosphorylation of p47^phox^ and its translocation to the plasma membrane. In addition, the extract inhibited MPO degranulation. In conclusion, by inhibiting both extracellular ROS production and neutrophil’s degranulation, *Anv* has strong anti-inflammatory properties which make it a promising candidate for further medicinal applications.

## Additional file

Additional file 1: Dose-effect of *Anvillea garcinii (Anv)*, *Zygophyllum gaetulum (Zyg)* and aspirin (Asp) on human neutrophils ROS production. Human neutrophils were incubated with increasing concentration of *Anv* (38–300 μg/mL), *Zyg* (38–300 μg/mL) or Asp (62–500 μg/mL) 15 min before stimulation with A) fMLF (10^−6^ M) or B) PMA (100ng/ mL). ROS was measured by luminol-amplified chemiluminescence and data are expressed as percentage to control (fLMF or PMA alone). All results are means ± SEM of three or more separate experiments. (DOC 35 kb)

## Abbreviations

*Anv*: *Anvillea garcinii* Coss. & Durieu
fMLF: N-formyl-methionyl-leucyl-phenylalanine
MPO: Myeloperoxidase
NOX2: Phagocyte NADPH oxidase
PMA: Phorbol myristate acetate
*Zyg*: *Zygophyllum gaetulum*
