# Supplementary material for: Anvillea garcinii extract inhibits the oxidative burst of primary human neutrophils
Source: BMC Complement Altern Med. 2016 Nov 3;16:433. doi: 10.1186/s12906-016-1411-7 (PMC5095960; doi:10.1186/s12906-016-1411-7)
Supplement: Additional file 1: — Dose-effect of Anvillea garcinii (Anv), Zygophyllum gaetulum (Zyg) and aspirin (Asp) on human neutrophils ROS production. Human neutrophils were incubated with increasing concentration of Anv (38–300 μg/mL), Zyg (38–300 μg/mL) or Asp (62–500 μg/mL) 15 min before stimulation with A) fMLF (10−6 M) or B) PMA (100ng/ mL). ROS was measured by luminol-amplified chemiluminescence and data are expressed as percentage to control (fLMF or PMA alone). All results are means ± SEM of three or more separate experiments. (DOC 35 kb) [file 12906_2016_1411_MOESM1_ESM.doc]

**Supplementary figure**


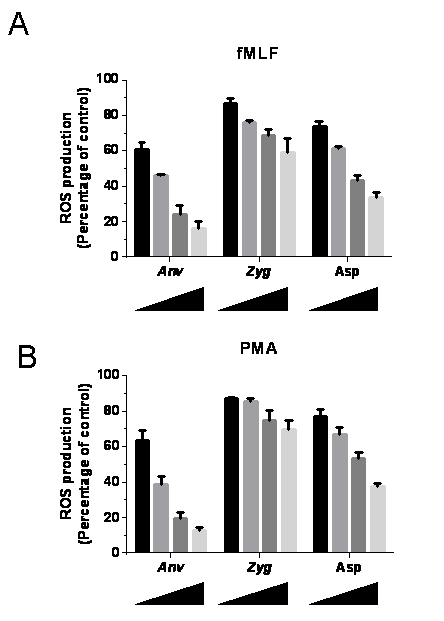


Supplementary Figure 1. Dose-effect of *Anvillea garcinii (Anv), Zygophyllum gaetulum (Zyg)* and aspirin (Asp) on human neutrophils ROS production. Human neutrophils were incubated with increasing concentration of *Anv* (38-300 μg/mL), *Zyg* (38-300 μg/mL) or Asp (62-500 μg/mL) 15 minute before stimulation with A) fMLF (10^-6^ M) or B) PMA (100ng/ mL). ROS was measured by luminol-amplified chemiluminescence and data are expressed as percentage to control (fLMF or PMA alone). All results are means ± SEM of three or more separate experiments.

.

**Supplementary Methods**

Measurement of ROS production by luminol-amplified chemiluminescence. ROS production was measured by the chemiluminescence method as described before; briefly, cells (5 x 10^5^) were resuspendend in 0.5 mL HBSS containing 10 μM luminol preheated to 37 °C in the thermostated chamber of the luminometer (Biolumat LB937; Berthold, Wildbad, Germany). Cells were incubated for 15 minutes with *Anv* (38-300 μg/mL), *Zyg* (38-300 μg/mL) or Asp (62-500 μg/mL) and then stimulated with fMLF (10^-6^ M) or PMA (100ng/ mL), and chemiluminescence was recorded.
